# Measuring Disability: Comparing the Impact of Two Data Collection Approaches on Disability Rates

**DOI:** 10.3390/ijerph120910329

**Published:** 2015-08-25

**Authors:** Carla Sabariego, Cornelia Oberhauser, Aleksandra Posarac, Jerome Bickenbach, Nenad Kostanjsek, Somnath Chatterji, Alana Officer, Michaela Coenen, Lay Chhan, Alarcos Cieza

**Affiliations:** 1 Chair of Public Health and Health Services Research, Department of Medical Informatics, Biometry and Epidemiology—IBE, Ludwig-Maximilians-University (LMU), Munich 81377, Germany; E-Mails: cornelia.oberhauser@med.lmu.de (C.O.); michaela.coenen@med.lmu.de (M.C.); 2 Social Protection and Labor, Human Development Network, The World Bank, Washington, DC 20433, USA; E-Mail: aposarac@worldbank.org; 3 Swiss Paraplegic Research, Nottwil 6207, Switzerland; E-Mail: jerome.bickenbach@paraplegie.ch; 4 Classification, Terminology and Standards, Department of Health Statistics and Informatics, World Health Organization, Geneva 1211, Switzerland; E-Mail: kostanjsekn@who.int; 5 Department of Health Statistics and Information Systems, World Health Organization, Geneva 1211, Switzerland; E-Mail: chatterjis@who.int; 6 Ageing and Life Course Unit, World Health Organization, Geneva 1211, Switzerland; E-Mail: officera@who.int; 7 National Institute of Statistics, Phnom Penh 12301, Cambodia; E-Mail: lay.chhan@gmail.com; 8 Blindness and Deafness Prevention, Disability and Rehabilitation (BDD), World Health Organization, Geneva 1211, Switzerland; E-Mail: acieza@who.int

**Keywords:** disability evaluation, International Classification of Functioning, Disability and Health, data collection, health surveys, disability surveys, screeners

## Abstract

The usual approach in disability surveys is to screen persons with disability upfront and then ask questions about everyday problems. The objectives of this paper are to demonstrate the impact of screeners on disability rates, to challenge the usual exclusion of persons with mild and moderate disability from disability surveys and to demonstrate the advantage of using an *a posteriori* cut-off. Using data of a pilot study of the WHO Model Disability Survey (MDS) in Cambodia and the polytomous Rasch model, metric scales of disability were built. The conventional screener approach based on the short disability module of the Washington City Group and the *a posteriori* cut-off method described in the World Disability Report were compared regarding disability rates. The screener led to imprecise rates and classified persons with mild to moderate disability as non-disabled, although these respondents already experienced important problems in daily life. The *a posteriori* cut-off applied to the general population sample led to a more precise disability rate and allowed for a differentiation of the performance and needs of persons with mild, moderate and severe disability. This approach can be therefore considered as an inclusive approach suitable to monitor the Convention on the Rights of Persons with Disabilities.

## 1. Introduction

Article 31 of the United Nations Convention on the Rights of Persons with Disabilities (CRPD) mandates that ratifying states “collect appropriate information, including statistical and research data, to enable them to formulate and implement policies to give effect to the present Convention” [1]. Currently, however, there is a lack of consensus on how to measure disability, and disability prevalence estimates are still strongly influenced by each country’s conceptual or legal definitions of disability and by the questions used to operationalize these definitions in disability, health and social surveys and censuses [2,3]. Such conceptual and definitional variability has not only an immediate impact on disability estimates, but may contribute in the middle and long run to inconsistent or insufficient policy solutions and, ultimately, negatively impact the lives of those experiencing disability. A recent review of disability surveys carried out in all world regions shows that the definition proposed in the WHO International Classification of Functioning, Disability and Health (ICF), as the outcome of “the interaction between an individual (with a health condition) and that individual’s contextual factors (personal and environmental factors)” [4], has been widely adopted in disability surveys over the past few years [5]. However, countries also tend to tailor this broad definition to their needs and to the goals of specific data collection efforts, such as surveys targeting eligibility for social programs. This might be done, for example, by introducing additional criteria specifying that a person will be considered disabled only if he or she experiences limitations that have lasted longer than one year. These country-specific additional criteria are usually operationalized in filter questions used to select participants for the survey and have an important impact on disability estimates and the comparability of these estimates across countries.

The conventional approach to collecting information on disability is to screen the population at the outset to identify ‘people with disabilities’ and then to ask this sub-population follow-up questions about everyday problems that they face. Screeners may either be impairment or functioning questions [2]. Impairment screeners are used to select a population of disabled individuals either by asking about the presence of an impairment or health condition, while functioning screeners target limitations in selected functioning domains because of a health condition, such as problems performing activities of daily living. The short set of questions developed by the Washington City Group (WG-6) as a general disability indicator for censuses and national surveys was not originally recommended to be used as a disability screener (WCG-Recommendations for the 2010 Round of Censuses). This set has, however, been frequently so used, as it is contains six standard basic functioning domains: seeing, hearing, walking or climbing steps, remembering or concentrating, washing all over or dressing and communicating [6]. The WG-6 was used to select respondents with disabilities in a series of surveys in several African countries about the living conditions of people with disabilities. Respondents answering at least two questions with “some difficulty” were included in the sample of disabled persons of the survey (http://www.sintef.no/home/projects/sintef-technology-and-society/2006/studies-on-living-conditions/).

One of the most troublesome issues with the standard approach of using screeners is that the suitability of resulting surveys to monitor the implementation of the CRPD is questionable. Article 1 states that “Persons with disabilities include those who have long-term physical, mental, intellectual or sensory impairments which in interaction with various barriers may hinder their full and effective participation in society on an equal basis with others” [1]. The CRPD definition stresses the rights of persons experiencing disabilities to participate in society on an equal basis with others, thus requiring an unbiased and direct comparison of the participation of persons with disabilities with the participation opportunities of the general population. This is not possible if only persons with disabilities are included in a disability survey.

In response to calls for improved disability data collection, the Model Disability Survey (MDS) project (http://www.who.int/disabilities/data/mds.pdf) was initiated by the WHO and the World Bank (WB) in 2011. The MDS is based on ICF and represents a revolution in the concept of disability measurement. The MDS conceptualizes disability as an outcome of interactions between a person with a health condition and environmental and personal factors, rather than just focusing on a person’s health state, impairments or functional limitations. As in the ICF, the MDS defines disability as a universal phenomenon experienced on a continuum from low to high levels of severity. The MDS utilizes a general population sample without screeners or filters. The aims of the MDS are to (1) achieve comparable and standardized prevalence estimates across countries, (2) provide the data needed to design appropriate interventions, programs and policies for persons with mild, moderate and severe levels of disability and (3) provide data needed to monitor the implementation of the CRPD by allowing for a direct comparison among people with disabilities of any level of severity and those without.

Although the shortcomings of screeners have been repeatedly reported, giving them up may be challenging for countries with a long tradition of using them. Analytically demonstrating the impact of using screeners on disability rates can support countries in their decision to move on to more inclusive strategies. Providing a direct comparison between people identified as disabled and those left out after applying a screener can call attention to the risk of excluding people in need from targeted interventions, programs and policies that could improve their life situations. Furthermore, attention has to be paid to those with mild and moderate levels of disability. Policy makers need to be made aware that that these persons are at risk of developing higher levels of disability and that their needs are as significant as those of the more easily-identifiable persons with severe disabilities. Interventions targeting this group with mild and moderate levels of disability would have, overall, a higher population impact than interventions for the severe only, measured in DALYs improvement across the population.

In the present work, we used the first pilot study of the WHO Model Disability Survey that was carried out in Cambodia to address three objectives:

(1) to analytically demonstrate the impact of disability screeners on disability rates,
(2) to challenge the usual *a priori* exclusion of persons experiencing mild and moderate levels of disability from disability surveys and
(3) to analytically demonstrate the advantage of using an *a posteriori* cut-off in a general population sample to identify persons experiencing disabilities.

## 2. Methods

### 2.1. Survey

In the implementation phase of the MDS, a pilot study was carried out in Cambodia in August 2014, which involved a convenience sample of 500 adults living in the Kampong Thom and Kampot provinces. Interviews were conducted in selected districts of these provinces to cover people living in both urban and rural areas. A quota sample aligned to match the final survey population was used because it was more feasible to implement then a probability sample and was considered adequate for a pilot test. The implemented Alpha version of the MDS comprises two questionnaires applied by trained interviewers. The household questionnaire is answered by the head of the household and has two modules: (1) the household roster, targeting a short description of the household and all household members; and (2) the children module, targeting disability and health conditions in children. The individual questionnaire is answered by a randomly-selected adult member of the household, takes between 60 and 120 minutes to be completed and has seven sections: (1) Section 1000: socio-demographic characteristics; (2) Section 2000: work history and benefits; (3) Section 3000: environmental factors; (4) Section 4000: functioning; (5) Section 5000: health conditions and capacity; (6) Section 6000: health-care utilization; and (7) Section 7000: satisfaction, personality and well-being. Only data from the individual questionnaire will be discussed in this paper. Section 5000 comprises questions targeting both difficulties in capacity, *i.e.*, the ways health problems affect how people function in multiple domains, and the presence of health conditions. The capacity questions included in this section encompass the Short Set of Questions on Disability (WG-6) proposed by the Washington City Group and include six domains: seeing, hearing, walking or climbing steps, remembering or concentrating, washing all over or dressing and communicating [6]. All questions in Section 4000 target performance, *i.e.*, how people actually function in multiple domains given health problems and the environmental barriers and facilitators that constitute their real-life situations. Response options of both capacity and performance items range from 1 (no difficulty or problem) to 5 (extreme difficulty or problem). The Alpha version of the MDS is available upon request (ciezaa@who.int).

### 2.2. Capacity and Performance Metric Scales

The MDS takes the approach that disability is a universal phenomenon characterized by a continuum ranging from low to high disability levels. This requires information on disability to be reported and analyzed using metric scales. According to the recommendations of the World Report on Disability (WRD), capacity and performance questions (Table 1) were used to develop a capacity and a performance scale, respectively, with metric properties.

**Table 1:** Items of the Model Disability Survey, Alpha version, used to build the performance and capacity metric scales.

**Section 4000, Functioning: Performance Questions**	
I4002	How much of a problem is standing for long periods such as 30 minutes for you?
I4003	How much of a problem is getting out of your home for you?
I4004	How much of a problem is walking a short distance such as a 100m for you?
I4005	How much of a problem is walking a kilometer for you?
I4006	How much of a problem is engaging in vigorous activities for you, such as **[add country specific examples]**?
I4007	How much of a problem is getting where you want to go for you?
I4009	How much of a problem is raising a 2 liter bottle of water from waist to eye level?
I4012	How much of a problem is toileting?
I4014	How much of a problem is looking after your health, eating well, exercising or taking your medicines?
I4015	How much of a problem do you have with seeing things at a distance?
I4017	How much of a problem do you have with hearing what is said in a conversation with another person in a quiet room?
I4019	How much of a problem is having pain for you?
I4020	How much of a problem do you have with sleep?
I4021	How much of a problem is feeling tired and not having enough energy?
I4023	How much of a problem do you have with coughing or wheezing?
I4025	How much of a problem do you have with felling worried, nervous or anxious?
I4026	How much of a problem is getting along with people who are close to you, including your family and friends?
I4030	How much of a problem is handling stress, such as controlling the important things in your life?
I4032	How much of a problem do you have with being understood, using your usual language?
I4035	How much of a problem is remembering to do the important things in your day to day life?
I4037	How much of a problem do you have with getting your household tasks done?
I4040	How much of a problem do you have with joining community activities, such as festivities, religious or other activities?
I4042	How much of a problem did you have with voting in the last elections?
I4043	How much of a problem do you have providing care or support for others?
I4045	*INTERVIEWER: If the respondent is currently not working, select the response option 98, not applicable.* How much of a problem is getting things done as required at work?
I4048	How much of a problem is using public or private transportation?
**Section 5000: Capacity questions**	
I5002	*INTERVIEWER: If I3019 = 1, then include [without glasses] in the question.* How much difficulty do you have seeing [without glasses]?
I5003	*INTERVIEWER: If I3023 = 1, then include [without* hearing aids*] in the question.* How much difficulty do you have hearing [without hearing aids]?
I5004	How much difficulty do you have walking or climbing steps because of your health?
I5005	How much difficulty do you have remembering or concentrating because of your health?
I5006	How much difficulty do you have washing all over or dressing because of your health?
I5007	Because of your health, how much difficulty do you have communicating, for example understanding or being understood using your usual (customary) language?
I5008	Because of your health, how much difficulty do you have doing things that require the use of your hands and fingers, such as picking up small objects or opening a container?
I5009	How much difficulty do you have sleeping because of your health?
I5010	How much difficulty do you have with shortness of breath because of your health?
I5011	How much difficulty do you have doing household tasks because of your health?
I5012	How much difficulty do you have providing care or support for others because of your health?
I5013	Because of your health, how much difficulty do you have with joining community activities, such as festivities, religious or other activities?
I5014	*INTERVIEWER: If the respondent is not working or receiving education, select the response option 98, not applicable.* How much difficulty do you have with your day to day work or school because of your health?
I5015	How much difficulty do you have with feeling sad, low or depressed because of your health?
I5016	How much difficulty do you have with feeling worried, nervous or anxious because of your health?
I5017	Because of your health, how much difficulty do you have getting along with people who are close to you, including your family and friends?
I5018	Because of your health, how much difficulty do you have coping with all the things you have to do?
I5019	How much bodily aches or pain do you have?

The partial credit model (PCM) was applied to develop the scales with metric properties [7,8]. The PCM, also called the polytomous Rasch model, is a unidimensional item response theory (IRT) model suitable for ordinal, polytomous items with which a latent scale is created [9]. Both persons and items can be located on the scale; for persons, the location is called ‘person ability’ and for items ‘item difficulty’. In addition, item thresholds are estimated for each item and indicate the locations on the latent trait where the item best discriminates among persons.

Model assumptions, namely unidimensionality, local independency and monotonicity, were evaluated *a priori*. Unidimensionality [10] was tested with bifactor analysis [11,12,13], which assumes the presence of a single general factor and multiple independent group factors. Two conditions must be met to assume underlying unidimensionality: (1) all items must load high on the general factor; and (2) factor loadings of items on the general factor must exceed those of the group factors. Permuted parallel analysis was applied to estimate the number of factors to be included in the bifactor analysis [14]. Bifactor analysis was applied on the polychoric correlation matrix [15,16], which is a measure of association between two latent continuous variables underlying two measured ordinal variables. Local independence [10] was examined based on residual correlations among items resulting from a single-factor factor analysis [17]. The PCM was estimated with and without the potential local dependent items showing residual correlations >0.25 to challenge how robust results were in the presence of question dependencies [18]. If item thresholds change significantly when considering local dependent items in the same model, all but one of them need to be excluded. Monotonicity was tested for each item by examining graphs of the item’s distribution of mean “rest-scores” [10], calculated for each person as the average raw score of all of the remaining non-missing items. Monotonicity can be assumed if persons with higher mean rest scores are consistently more likely to have more problems in the selected item.

If unordered thresholds were observed when fitting the PCM, the response options of such items were collapsed until all thresholds were in the correct order. Differential item functioning (DIF) was tested for gender and age groups using iterative hybrid ordinal logistic regression with the change in McFadden’s pseudo R-squared measure (above 0.02) as a DIF criterion [19,20] to examine if males and females, as well as persons from different age groups with the same (latent) disability level have different probabilities of giving a certain response to an item. If an item shows DIF, the item must be split into separate items for the groups and the model re-estimated.

To examine whether the items fit the PCM, (unweighted) outfit and (weighted) infit mean squares were calculated [21]. Values close to 1 indicate good item fit, while values ‘much’ larger than 1 indicate underfit (*i.e*., the observed data vary much more than expected by the model: a violation of the model), and values ‘much’ smaller than 1 indicate overfit (*i.e*., the data vary much less than expected by the model) [8,21]. The majority of studies employ a range of 0.7 to 1.3. Persons’ disability level was linearly transformed to scales ranging from 0 (lowest level of disability) to 100 (highest level of disability).

### 2.3. Disability Rates

Two approaches to estimating disability rates were compared in the present study to analytically demonstrate the impact of disability screeners on these rates. In the first approach, we applied a conventional *a priori* disability functioning screener to define the “disabled” population. In the second approach, we applied the cut-off method used by WHO in the WRD to identify the “disabled” population *a posteriori* in a general population sample.

The *a priori* disability functioning screener is based on the WG-6 questions:

- How much difficulty do you have seeing [without glasses]?
- How much difficulty do you have hearing [without hearing aids]?
- How much difficulty do you have walking or climbing steps because of your health?
- How much difficulty do you have remembering or concentrating because of your health?
- How much difficulty do you have washing all over or dressing because of your health?
- Because of your health, how much difficulty do you have communicating, for example understanding or being understood using your usual (customary) language?

Although the Washington City Group explicitly states that the WG-6 “are not intended as disability screening questions unless it is clearly understood that some persons of interest, such as those with learning or psychological disabilities, will not be appropriately included in the identified population” (WCG-Recommendations for the 2010 Round of Censuses), their questions have been used as a screening instrument in several surveys for practical purposes. For census tabulation, the Washington City Group recommends that the group of disabled persons should include everyone with at least one domain described as with “a lot of difficulty” or “cannot do it at all” (WCG-Recommendations for the 2010 Round of Censuses). Since we evaluated survey data in our present work, not census data, we decided to adopt criteria already used in surveys and selected the criteria applied to the Living Conditions Surveys carried out in several countries in Africa [22], namely that people with at least two questions answered with “some difficulty” are considered as “disabled”.

The cut-off used to identify the sample of “disabled” *a posteriori* is the one proposed in the WRD. In the WRD, a metric of functioning ranging from 0 (no functioning difficulty) to 100 (complete functioning difficulty) was constructed based on capacity questions, and the mean score on this metric of the following two groups (combined) was used to set a meaningful threshold for significant disability:

(1) Persons reporting at least extreme difficulties in at least one of eight functioning domains, namely:
  - Mobility (moving around and vigorous activity)
  - Self-care (self-care, appearance, grooming)
  - Pain (bodily aches and pains, bodily discomfort)
  - Cognition (concentrating, remembering, learning)
  - Interpersonal relationships (participation in the community, dealing with conflicts)
  - Vision (distance vision, near vision)
  - Sleep and energy (falling asleep, feeling rested)
  - Affect (feeling depressed, worry, anxiety)
(2) Persons reporting (at least) one of four chronic conditions likely to lead to disability, namely:
  - Asthma or breathing problems
  - Diabetes
  - Arthritis
  - Depression

In the WRD, the meaningful threshold point separating persons with significant disability from other respondents was around 40. In the present work, the cut-off identifying persons with significant disability was estimated in the same manner using the capacity metric as the reference. Items used to operationalize the eight functioning domains were: I5004, I5006, I5019, I5005, I5013, I5017, I5002, I5009, I5015 and I5016 (Table 1). At least extreme difficulties in any of these items was defined as 4 or 5 in the response scale. The cut-offs for mild and moderate disability were set based on the sample distribution. Persons with no disability mentioned no health conditions and no difficulties in any capacity domain.

The first and second approaches will hereafter be referred to as “screener approach” and “WRD approach”, respectively.

### 2.4. Comparison of Disabled versus Non-Disabled Individuals

Comparisons using descriptive statistics were carried out to challenge the common exclusion of persons experiencing mild and moderate levels of disability from disability surveys, as well as to analytically demonstrate the advantages of using an *a posteriori* cut-off in a general population sample.

The following samples were compared:

(I) “disabled” *versus* “non-disabled” using the *a priori* screener,
(II) samples with high, moderate, mild and no levels of disability defined using the cut-off.

These samples were compared using descriptive statistics regarding:

(1) Performance levels (performance metric),
(2) Sociodemographic aspects (age, gender and Table 2, Block 1),
(3) Hindering and facilitating aspects of the environment (Table 2, Block 2),
(4) Quality of life (Table 2, Block 3).

**Table 2:** Items of the Model Disability Survey, Alpha version, used to exemplarily compare samples of persons classified as “disabled” and “non-disabled”, as well as persons classified as having mild, moderate or severe levels of disability.

**Block 1**	**Work and Education**
I1014	What is the highest level of education that you have completed?
I2005	What is your current working situation?
**Block 2**	**Hindering and Facilitating Aspects of the Environment**
I am going to ask you some general questions about your environment. I would like to know if the environment makes it easy or hard for you to do the things you need or want to do. I want you to answer the following questions on a scale from 1 to 5, where 1 means very easy and 5 means very hard, shown on show card 002.	
I3001	To what extent does your workplace or school make it easy or hard for you to do the things you need or want to do at work or school?
I3002	To what extent do the health facilities you need to use regularly make it easy or hard for you to use them?
I3003	To what extent do the places where you want to or need to socialize and engage in community activities make it easy or hard for you to do this?
I3004	How easy or hard do the shops, banks and post office in your neighborhood make it for you to do things you need or want to do?
I3005	To what extent do your regular places of worship make it easy or hard for you to worship?
I3006	To what extent does the transportation you need or want to use make it easy or hard for you to use it?
I3007	How easy or hard does your dwelling (including toilet and all rooms) make it for you to do things you need or want to do?
I3008	How easy or hard does your natural environment of the place you usually live—its temperature, terrain, and climate—make it for you to do things you need or want to do?
I3009	How easy or hard does the lighting, noise, and crowds, in your surroundings make it for you to do things you need or want to do?
**Block 3**	**Quality of Life**
I7001	In the past 30 days, how would you rate your quality of life?

These areas were selected for their core importance in countries monitoring the CRPD.

The complete data analysis was performed with R Version 2.15.2 [23].

## 3. Results

### 3.1. Sample

Data from all 500 interviewed adults were analyzed. The mean (standard deviation) age of respondents was 42.81 (SD 14.15). Sociodemographic characteristics are presented in Table 3.

**Table 3:** Sociodemographic characteristics of the sample used in the study.

		No. of Persons	%
**Gender**	Male	193	38.6
	Female	307	61.4
**Marital Status**	Never married	44	8.8
	Married	368	73.6
	Cohabiting	24	4.8
	Separated/divorced	8	1.6
	Widowed	56	11.2
**Education Level**	No schooling or no grade completed	95	19.0
	Elementary education	214	42.8
	Vocational education	5	1.0
	Secondary school	170	34.0
	University	13	2.6
	Post-graduate studies	3	0.01
**Work Situation**	Not working	29	5.8
	Working for wages or salary with an employer (full- or part-time)	79	15.8
	Working for wages, currently on sick leave >3 months	6	1.2
	Self-employed or own-account worker	270	54.0
	Working as unpaid family member	50	10.0
	Retired due to age	5	1.0

### 3.2. Capacity Metric

**Figure 1:**
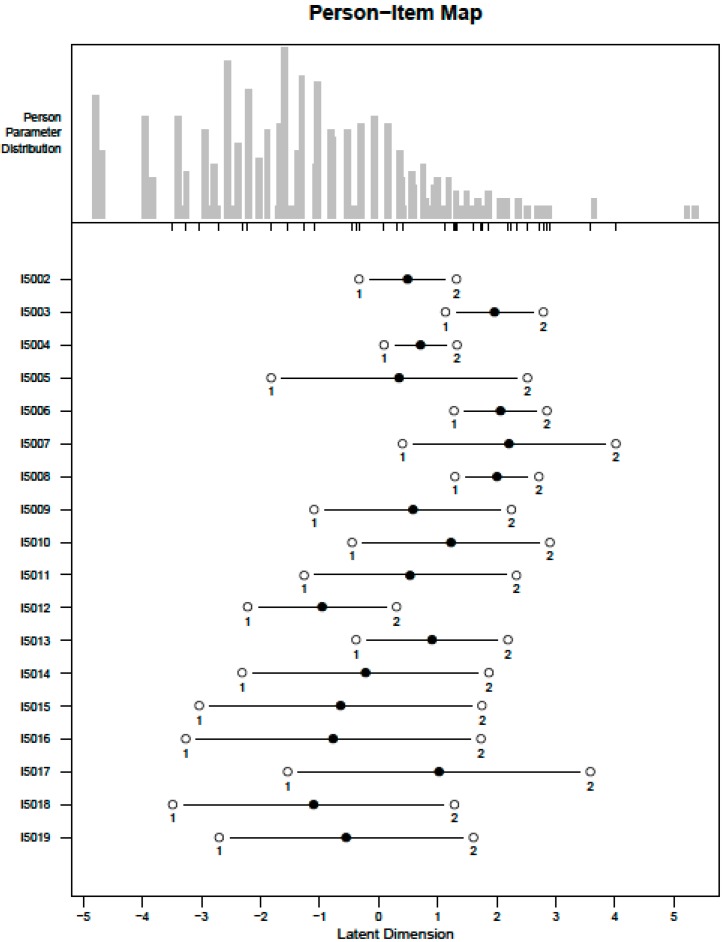
Person-item map of the capacity metric built with Rasch analyses.

When testing the IRT model assumptions, permuted parallel analysis indicated the presence of two factors. In the bifactor analysis, the general factor accounted for 55% of the overall variance, which makes up 83% of the variance explained by the factor model. The loadings of the general factor (ranging from 0.58 to 0.89) exceeded those of the group factors for all items, supporting the assumption of unidimensionality. Two residual correlations exceeded 0.25, those between the items on seeing and hearing (0.285) and between the items “feeling sad, low or depressed” and “feeling worried, nervous or anxious” (0.332). All other items already fulfilled the assumption of local independency. Monotonicity was graphically confirmed for all items. When fitting the PCM, the thresholds of seven items were initially disordered. Therefore, all items were collapsed into 01122, resulting in ordered thresholds (Model 1). None of the items showed DIF by gender, while four items (seeing, hearing, walking or climbing steps and feeling sad, low or depressed) showed DIF by age groups. With regard to item fit, all but two items (seeing and hearing) fit the model according to both their outfit and infit mean squares. Only these two had too large outfit mean squares exceeding 1.3 (2.03 and 1.37), but acceptable infit mean squares below 1.3 (1.28 and 1.1). Sensitivity analyses were performed with regard to local independency and DIF. When omitting ‘hearing and feeling worried’ and ‘nervous and anxious’ (Model 2), person’s abilities hardly changed (Pearson correlation 0.996). When splitting the four DIF variables by age groups (Model 3), person’s abilities again barely changed (Pearson correlation 0.999). We therefore decided to stick to Model 1 despite these violations of the model assumptions. The person-item map is shown in Figure 1.

**Figure 2:**
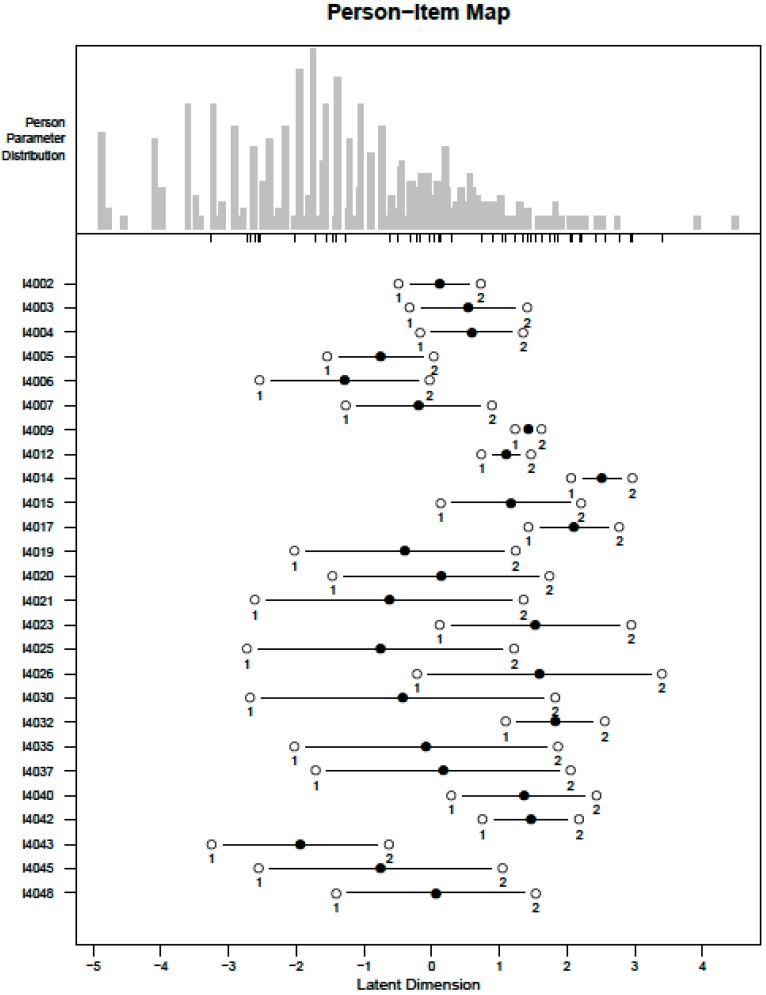
Person-item map of the performance metric built with Rasch analyses.

### 3.3. Performance Metric

The following 18 ICF domains covered in Section 4000 were used to build a performance scale: mobility, hand and arm use, self-care, seeing, hearing, pain, energy and drive, breathing, affect, interpersonal relationships, handling stress, communication, cognition, household tasks, community participation, citizenship participation, caring for others, work and schooling, and transportation (items in Table 1). Permuted parallel analysis indicated the presence of three factors. In the bifactor analysis, the general factor accounted for 49% of the overall variance, which makes up 78% of the variance explained by the factor model. The loadings of the general factor (ranging from 0.44 to 0.92) exceeded those of the group factors for all items, supporting the assumption of unidimensionality. Five residual correlations slightly exceeded 0.25 (0.33 for I4025 with I4030, 0.27 for I4025 with I4035, 0.33 for I4026 with I4032, 0.25 for I4040 with I4042 and 0.28 for I4037 with I4045). However, excluding a subset of these items would involve excluding ICF domains, and residual correlations just slightly exceeded 0.25; all of them were kept in the model. All other items fulfilled the assumption of local independency. Monotonicity was graphically confirmed for all items. All items on performance were collapsed into 01122, resulting in ordered thresholds (Model 1). None of the items showed DIF by gender, while one item (I4015) showed DIF by age. However, the item could not be split for age groups, as the frequencies for large problems were too low. With regards to item fit, all but six items fit the model according to both their outfit and infit mean squares. The person-item map is shown in Figure 2. The Pearson correlation of the performance metric resulting from this reduced item set compared to the model with all items in Section 4000 was 0.972.

### 3.4. Disability Rates

The screener approach based on WG-6 led to a disability rate of 32.2%, *i.e.*, 32.2% of the respondents answered at least two questions ‘with some difficulty’.

The estimated threshold point for the *a posteriori* cut-off separating persons with significant disability from other respondents was 47.4. This cut-off led to a disability rate of 25.7%, *i.e.*, 25.7% of the sample experiences important disability. Using the distribution of the capacity score (Figure 1), persons with scores below 30 were considered to have mild disability, while persons with scores between 30 (≥30) and 47.4 were considered to have moderate disability. Persons with no problem in any capacity questions and no health conditions were classified as having no disability.

The overlap between the two approaches is shown in Table 4. The screener approach correctly identified 78% of all persons with severe disabilities and about 30% with moderate disabilities, who are then all classified as disabled without further differentiation. As such screeners are used to select the respondents for a survey, in the present example, that would mean that 22% of persons with severe disabilities, 71% of persons with moderate disabilities and almost all persons reporting mild disabilities would have been excluded from the survey. Using the screener approach, 161 persons would have been selected for the survey, although the metric scale points out that altogether, 472 persons experienced disabilities to different extents.

**Table 4:** Comparison of the person identified as disabled *vs*. not disabled with the screener approach and the persons in the severe, moderate, mild and no disability groups with the World Report on Disability (WRD) approach. WG-6, Short Set of Questions on Disability, Washington City Group.

	Functioning Screener WG-6		
Cut-off WRD Approach	Not disabled	Disabled	Total
**Severe**	28 (22%)	100 (78%)	128 (100%)
**Moderate**	141 (71%)	59 (29%)	200 (100%)
**Mild**	142 (99%)	2 (1%)	144 (100%)
**No disability**	27 (100%)	0	27 (100%)
**Total**	**338 (68%)**	**161 (32%)**	**499 (100%)**

### 3.5. Comparison of Disabled *versus* Non-Disabled Individuals

#### 3.5.1. Performance Levels (Performance Metric)

**Figure 3:**
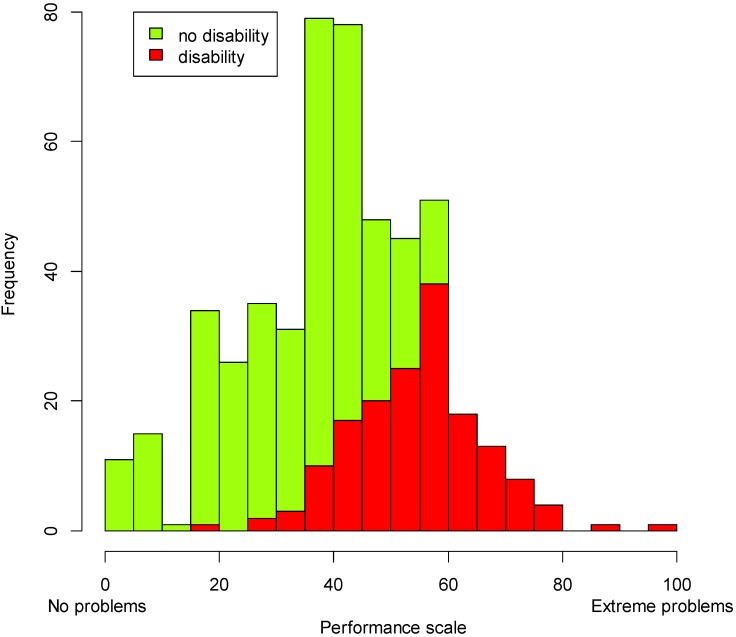
Distribution of the sample on the performance scale; groups generated with the screener approach.

Since the performance questions concern how people actually function in multiple domains in light of their health problems and the environmental barriers and facilitators they face, the performance scale reflects the extent to which people with disabilities live their lives and participate in society and is essential when monitoring the CRPD. The histogram generated with the screener approach (Figure 3) shows in red the population that we would be looking at using the screener as a filter. For this country, what we see is that the majority of the “disabled” sample experiences high levels of problems in performance.

**Figure 4:**
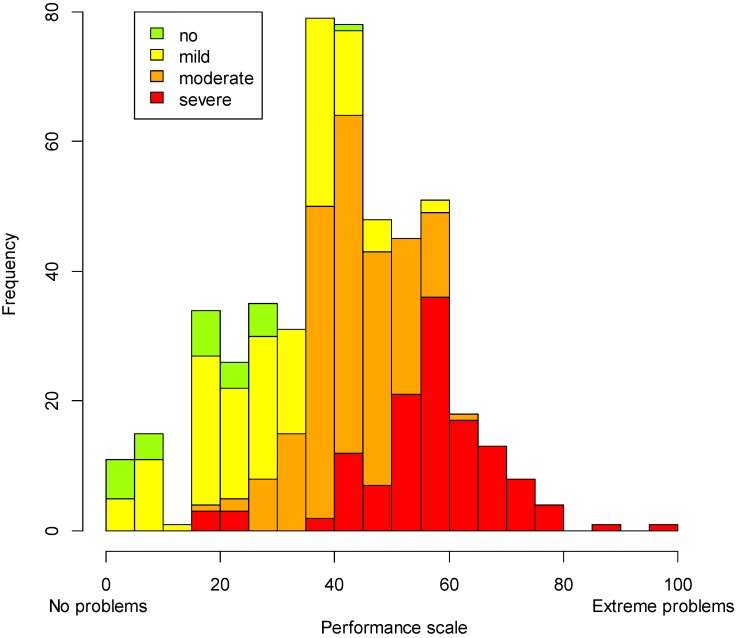
Distribution of the sample on the performance scale; groups generated with the WRD approach.

The histogram generated from the WRD approach (Figure 4) shows a similar distribution of performance for persons with severe disabilities (red), but even in this group, there is variability in the level of performance. Based on our understanding of performance, *i.e*., how people actually function in multiple domains given health problems and the environmental barriers and facilitators that constitute their real-life situations, we hypothesize that this variation might be associated with differences in environmental factors, such as receiving adequate treatment, accessibility to assistive devices or support provided by the family. The histogram additionally shows the level of performance of persons with moderate (orange) and mild (yellow) disability levels, which are already worse than the performance levels of persons living in the country without disabilities (green) who serve as a reference group to check (as requested in the CRPD) if persons with disabilities carry out their lives and participate in society on an equal basis with others.

#### 3.5.2. Sociodemographic Aspects

Table 5 provides a comparison of the two approaches with respect to gender, age, education and work. The table shows that, in all of these aspects, the disabled population selected using the screener approach comes very close to the population identified as having severe disabilities using the WRD approach, although it includes about 30% of persons with moderate disabilities. Using the WRD approach and the four generated groups, including persons without a disability, a higher level of differentiation is achieved, and direct comparisons are made possible. Direct comparisons highlight, for instance, that while the majority of persons with severe disabilities are in the age group from 39.5 to 59.5 years old, the majority of persons with mild disabilities are younger and in the age range from 17.5 to 39.5 years of age. The table also highlights meaningful differences between the groups of non-disabled persons following the screener approach and the WRD approach. Taking education as an example, figures estimated with the screener approach show that the majority of non-disabled persons have completed elementary school, while figures estimated with the WRD approach show that the majority of non-disabled persons have completed secondary school. The lower level of education observed in the first estimate could result from the inclusion of people with mild and moderate disability levels in this group.

**Table 5:** Comparison of the functioning screener approach and the WRD approach regarding gender, age, education and work.

		Functioning Screener WG-6		Cut-off WRD Approach			
	*N*	Not Disabled *N* = 338	Disabled *N* = 161	No Disability *N* = 27	Mild Disability *N* = 144	Moderate Disability *N* = 200	Severe Disability *N* = 128
**Gender**							
Male (%)	193	36.87	42,24	51.85	40.28	34.5	40.62
Female (%)	307	63.13	57.76	48.15	59.72	65.5	59.38
**Age (Ranges)**							
17.5 to 39.5 years old (%)	226	56.42	23.27	77.78	58.04	44.67	26.98
39.5 to 59.5 years old (%)	200	37.01	47.8	18.52	36.36	47.21	39.68
59.5 to 100 years old (%)	68	6.57	28.93	3.7	5.59	8.12	33.33
**Education**							
No schooling (or never completed any grade) (%)	95	13.57	30.43	3.7	13.19	17	32.03
Elementary education (%)	214	42.18	44.1	25.93	38.89	48	42.19
Secondary school (%)	170	39.23	22.98	62.96	42.36	31	23.44
Other school level completed * (%)	21	5.01	2.48	7.41	5.56	4	2.34
**Work**							
Currently not working (%)	29	4.7	11.67	3.7	4.51	4.4	14.43
Working for wages or salary with an employer (%)	79	18.5	16.67	29.63	15.79	19.23	15.46
Self-employed or own-account worker (%)	270	63.64	55.83	66.67	65.41	62.09	53.61
Working as unpaid family member (%)	50	11.91	10	0	13.53	12.09	10.31
Other working situation** (%)	11	1.25	5.83	0	0.75	2.2	6.19

* Vocational education, university, post-graduate degree; ** sick leave, retired by age.

#### 3.5.3. Hindering and Facilitating Aspects of the Environment

**Figure 5:**
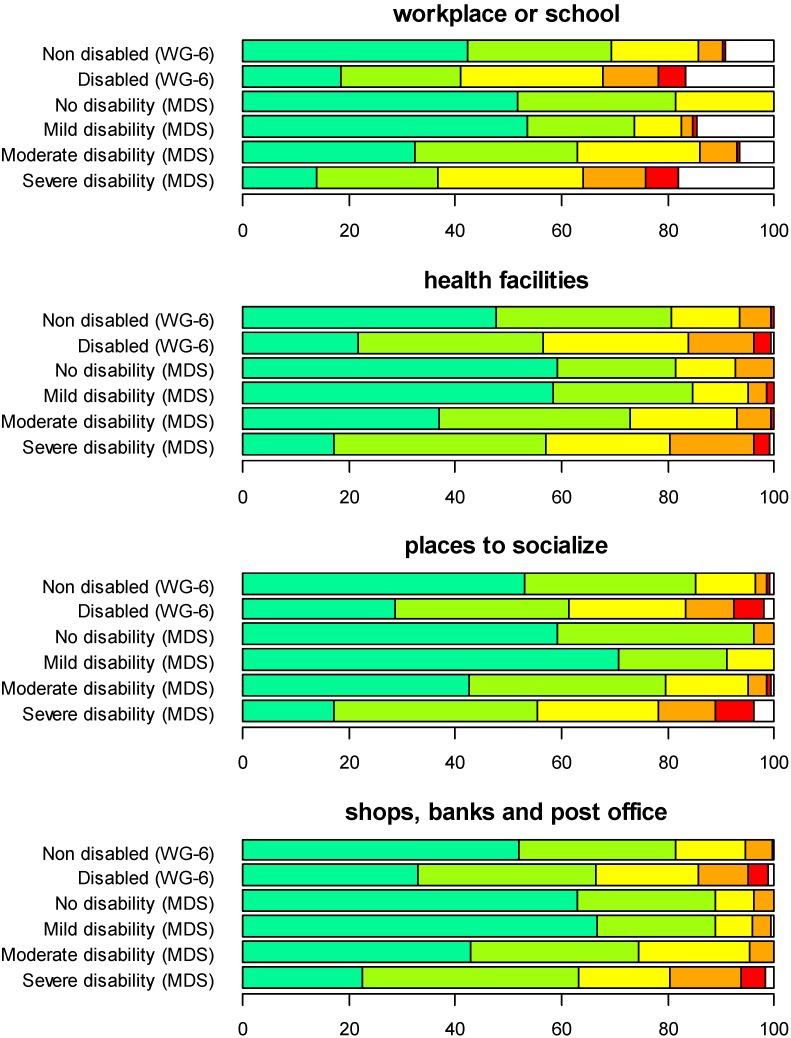
Comparison of the samples generated with the functioning screener approach and the WRD approach for several aspects of the environment that might be hindering and facilitating, on a scale from one to five, where one (dark green) means very easy and five means very hard (red). Dark green: very easy; light green: easy; yellow: neither easy nor hard; orange: hard; red: very hard. MDS, Model Disability Survey.

**Figure 6:**
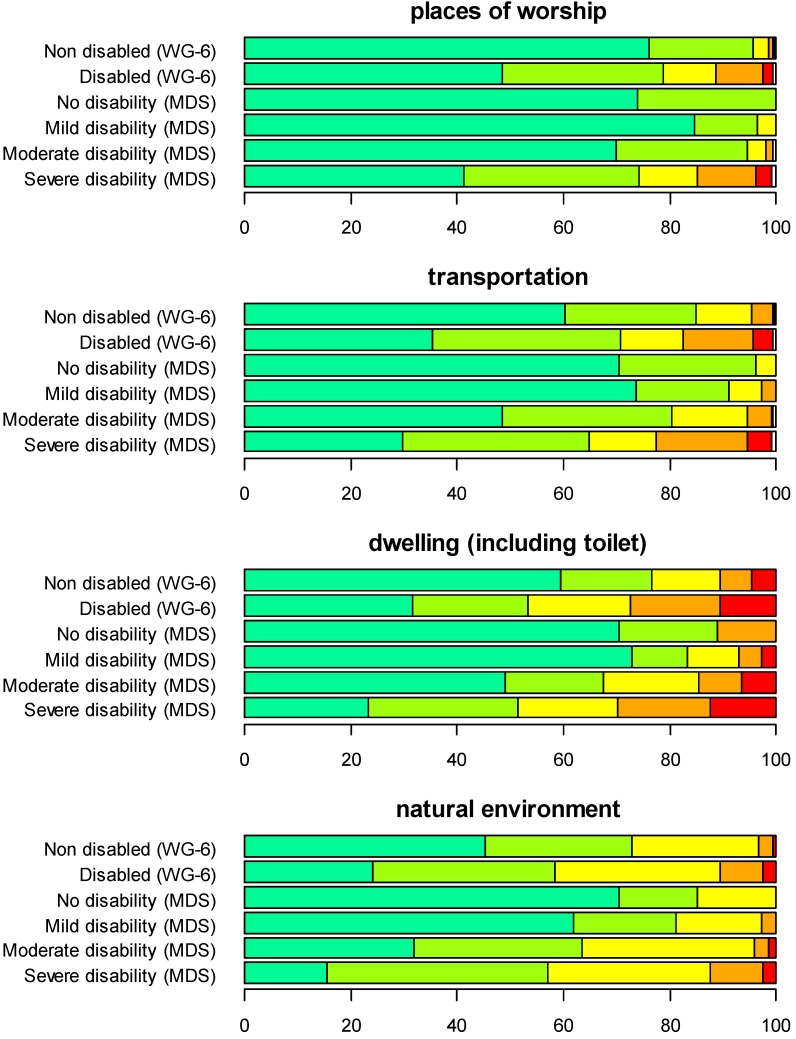
Comparison of the samples generated with the functioning screener approach and the WRD approach for several aspects of the environment that might be hindering and facilitating, on a scale from one to five, where one (dark green) means very easy and five means very hard (red). Dark green: very easy; light green: easy; yellow: neither easy nor hard; orange: hard; red: very hard.

Figure 5 and Figure 6 show a comparison of the two approaches for several environmental aspects that might prove to be hindering or facilitating on a scale from one to five, whereby one (dark green) indicates ‘very easy’ and five indicates ‘very hard’ (red).

In both approaches and for almost all aspects, persons considered non-disabled experience the environment as hindering to different extents and answer four (hard, orange) or five (very hard, red). In the screener approach, this probably arises from the fact that people experiencing different levels of disability are still included in the strata, while in the WRD approach, this reflects the conventional approach in the individual countries and could be used as a reference group. Taking the example of health facilities, in both approaches, about 7% of the non-disabled people experienced access to health facilities as a hindrance. In the WRD approach, we could conclude that accessibility to health facilities was already a problem for the general population and seemed to become even worse for persons with disabilities, endorsing the response options hard (orange) or very hard (red) in a manner in which a dose-response relationship between disability level and the accessibility to health facilities could be hypothesized. A similar pattern could be observed for other areas, as well. It is important to stress that persons with mild disabilities already seem to experience problems in the environment, for instance in transportation and within their own dwellings.

#### 3.5.4. Quality of Life

**Figure 7:**
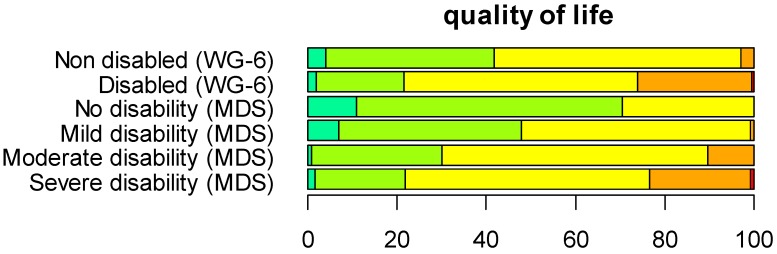
Comparison of the samples generated with the functioning screener approach and the WRD approach for quality of life. Dark green: very good; light green: good; yellow: neither poor nor good; orange: poor; red: very poor.

The quality-of-life rating again shows that the results obtained with the screener approach for the disabled correspond to the sample with severe disabilities. Results obtained with the WRD approach point out a dose-response relationship between disability level and quality of life (Figure 7).

## 4. Discussion

In an effort to support the use of disability data collection strategies suitable for monitoring the CRPD, this study aimed to analytically demonstrate both the impact of disability screeners on disability rates and the advantage of using an *a posteriori* cut-off in a general population sample to identify persons experiencing disabilities. It is important to stress, however, that we used data from a pilot study of the Model Disability Survey in Cambodia, which included a non-representative, convenience sample of 500 persons. Consequently, reported disability rates are exemplary estimates for this convenience sample and must not be taken as official disability rates for the country. Our results show that disability screeners, *i.e.*, screeners applied to a population at the outset to identify ‘people with disabilities’ for a survey, lead to imprecise disability rates and failed to correctly identify more than 20% of persons with severe disabilities. Importantly, this procedure leaves about 70% of persons already experiencing moderate levels of disability and nearly all persons reporting mild disability levels out of disability surveys. The use of an *a posteriori* cut-off and a general population sample leads to a more precise estimation of the prevailing rate of severe disability. This approach also discloses the magnitude of performance problems experienced by persons with mild and moderate disabilities and, therefore, challenges the usual *a priori* exclusion of these persons from disability surveys used to monitor the implementation of the CRPD.

A fundamental difference between the two approaches is their underlying assumption on how difficulties in functioning domains should translate into disability rates. The screener approach assumes that difficulties in different functioning domains are directly comparable, *i.e.*, that persons stating they have, for instance, important difficulties in communicating and hearing or important difficulties in hearing and walking have the same level of disability. Both persons would be classified as “disabled”. According to this assumption, a disability rate is estimated by simply counting the percentage of persons fulfilling a certain criterion, like the number of persons with important difficulties in at least two functioning domains.

Our psychometric analyses do not support this assumption. The item threshold for important difficulties in communicating is higher than for important difficulties in hearing, cognition and washing, which are, in turn, higher than those for important difficulties in seeing or walking. This means that persons with important difficulties in communicating are expected to have a severe level of disability (and difficulties in other domains), while those with important difficulties in hearing, cognition and washing, but not in communicating, are expected to have moderate levels of disability, and those with important difficulties just in seeing and/or walking are expected to have rather mild levels of disability. Wrongly assuming that important difficulties in at least two functioning domains is sufficient to decide that the level of disability a person experiences is severe might be one reason why the screener approach generated a disability rate of over 32%. In fact, this rate classifies 30% of persons with moderate disabilities erroneously as severely disabled.

Countries applying this rather easy and straightforward screener approach must be aware of the high risk of generating an overestimated, yet imprecise, rate of disability and of its consequences for policy making. The example of Brazil is instructive. In its 2010 census, Brazil included the WG-6 for the first time with the result that an impressive disability rate of 23.9% was estimated, *i.e.*, 45.6 million people were classified as disabled [24]. In the 2000 census, the disability rate had been 14.5%. In 2010, the highest prevalence of disability was seen, with 18.8% of the population claiming vision difficulties, probably because a large proportion of the population in Brazil had no access to an essential assistive device: glasses. As we demonstrate in the present work, however, persons with important difficulties in seeing are expected to have mild to moderate levels of disability, and in the case of Brazil, many might require only a simple public health intervention, such as the provision of glasses. The appropriateness of such high and not further differentiated disability rates can, therefore, be questioned in light of its consequences for intervention planning, policy making and the allocation of health and social resources to meet the needs of persons with different levels of disability.

The approach recommended in the WRD to estimate a disability rate is fundamentally different, as it uses questions of functioning domains to first create a scale of disability with metrical properties and then combines the average disability score of persons with disabling health conditions and the average disability score of persons with important problems in eight functioning domains to define the rate of persons with severe levels of disability. In other words, the WRD approach acknowledges that people with important difficulties in different functioning domains may have significantly different levels of disability associated with specific levels of performance and specific needs regarding interventions. The WRD approach also assumes that disability is a universal phenomenon characterized by a continuum ranging from low to high disability levels. As a consequence, disability must be measured by creating a scale with metric properties that integrates information about functioning domains and takes into account their differences. Our data show, for instance, that persons with important vision problems (but no problems in hearing, washing and communicating) are expected to have mild to moderate disability levels. As a consequence, these people would not be included in the group experiencing severe disabilities, as was the case in Brazil, and more precisely targeted interventions could be developed.

The WRD approach is initially more challenging, as it involves more time and personnel resources to recruit a general sample, to run a comprehensive disability survey like the MDS and to perform elaborate statistical analyses; but the approach pays off in terms of providing precise information about the percentage of the population experiencing significant disability and in terms of its usefulness in monitoring the implementation of the CRPD. By using a general population sample, this approach goes beyond defining disability in terms of having or not having a disability to that of providing countries with differentiated information about the level of performance and the specific needs of the population experiencing mild, moderate and severe levels of disability. Finally, it also provides a picture of the situation of persons without a disability, which can be set as baseline data providing an unbiased picture of the extent to which the environment in a country hinders or facilitates a person, even in the absence of a health condition.

This study should be understood in light of its limitations. Data used in the present work were obtained in a pilot study including a non-representative, convenience sample. Consequently, the group of persons without a disability was very small. Moreover, the criteria used as the disability screener are some of the many possible. Different criteria would have led to different disability rates. We, however, selected criteria that have often been used and that reflect the common practice in the field.

## 5. Conclusions

In an effort to support the use of disability data collection strategies suitable to monitor the CRPD, we showed that disability screeners lead to imprecise disability rates and to the exclusion of persons with mild to moderate disability levels from disability surveys, although these already experience important problems in daily life, have different needs than persons with severe disabilities and are at risk of deteriorating. It is this population that would greatly benefit from interventions that reduced the risk of developing more severe disabilities during life. The use of an *a posteriori* cut-off and a general population sample, which is the approach recommended in the WRD, leads to lower, but more precise rates of severe disability, allows for a differentiation of the needs of persons with mild, moderate and severe levels of disability and for a direct comparison between them and the population without disabilities. The WRD approach is, therefore, an inclusive approach suitable to monitor the implementation of the CRPD and to deliver the data needed to plan and design targeted disability interventions.
